# Supplementary material for: Time‑resolved multi-omic analysis of paclitaxel exposure in human iPSC‑derived sensory neurons unveils mechanisms of chemotherapy‑induced peripheral neuropathy
Source: Cell Death Dis. 2026 Feb 10;17(1):211. doi: 10.1038/s41419-026-08445-2 (PMC12921266; doi:10.1038/s41419-026-08445-2)
Supplement: Supplementary file 15 — Dataset 1. [file 41419_2026_8445_MOESM15_ESM.zip › CDD_Open_Data/04_RNA_Seq/Timeline_panel_plots_etc_2023-01-23_1609.zip!/Panel_Plots_etc/01_contrasts_tmod_panel_plot_tmod_pval_filter_0.05_AUC0.5_L2FC0.5.pdf]

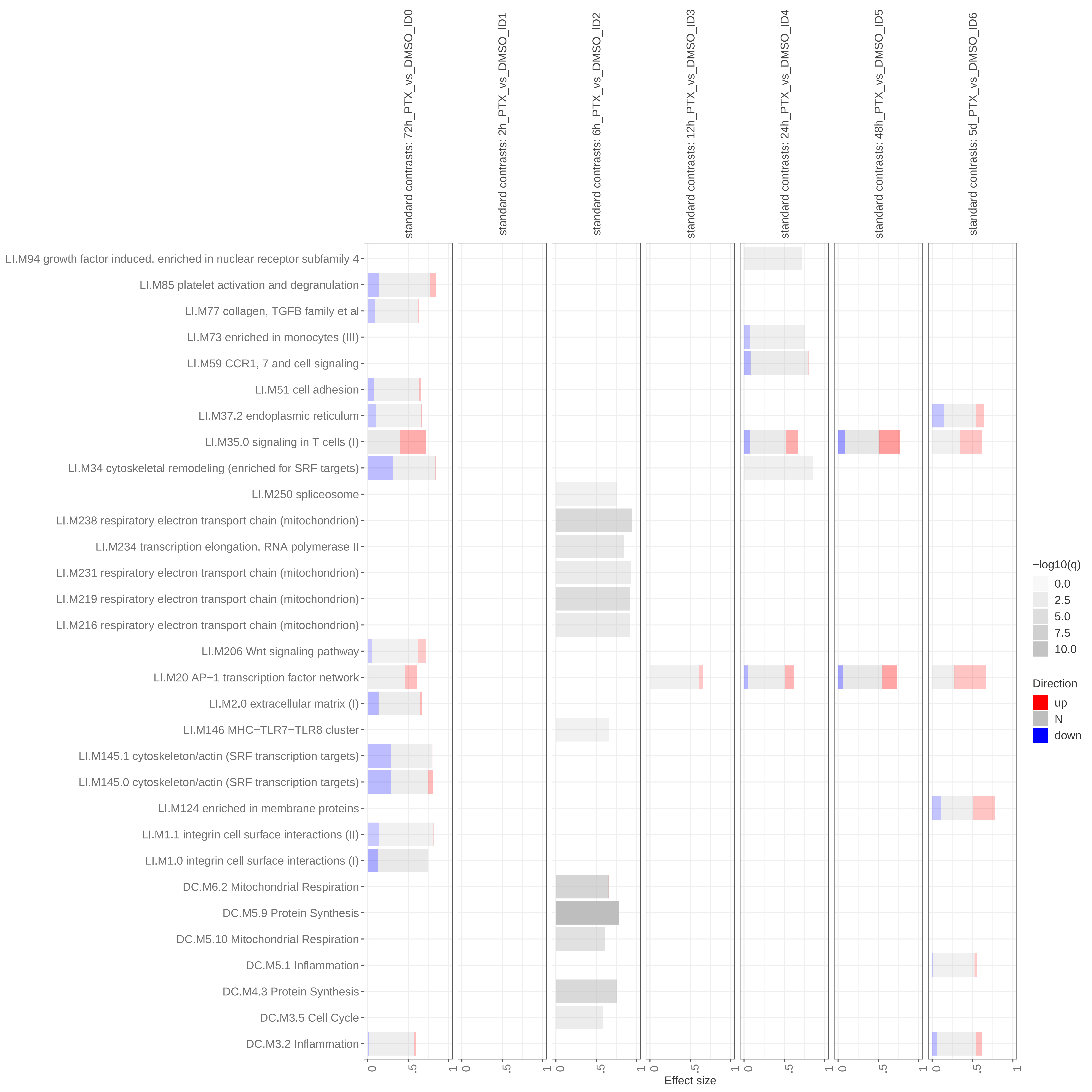

standard contrasts: 72h_PTX_vs_DMSO_ID0
standard contrasts: 2h_PTX_vs_DMSO_ID1
standard contrasts: 6h_PTX_vs_DMSO_ID2
standard contrasts: 12h_PTX_vs_DMSO_ID3
standard contrasts: 24h_PTX_vs_DMSO_ID4
standard contrasts: 48h_PTX_vs_DMSO_ID5
standard contrasts: 5d_PTX_vs_DMSO_ID6
LI.M94 growth factor induced, enriched in nuclear receptor subfamily 4
LI.M85 platelet activation and degranulation
LI.M77 collagen, TGFB family et al
LI.M73 enriched in monocytes (III)
LI.M59 CCR1, 7 and cell signaling
LI.M51 cell adhesion
LI.M37.2 endoplasmic reticulum
LI.M35.0 signaling in T cells (I)
LI.M34 cytoskeletal remodeling (enriched for SRF targets)
LI.M250 spliceosome
LI.M238 respiratory electron transport chain (mitochondrion)
LI.M234 transcription elongation, RNA polymerase II
LI.M231 respiratory electron transport chain (mitochondrion)
LI.M219 respiratory electron transport chain (mitochondrion)
LI.M216 respiratory electron transport chain (mitochondrion)
LI.M206 Wnt signaling pathway
LI.M20 AP−1 transcription factor network
LI.M2.0 extracellular matrix (I)
LI.M146 MHC−TLR7−TLR8 cluster
LI.M145.1 cytoskeleton/actin (SRF transcription targets)
LI.M145.0 cytoskeleton/actin (SRF transcription targets)
LI.M124 enriched in membrane proteins
LI.M1.1 integrin cell surface interactions (II)
LI.M1.0 integrin cell surface interactions (I)
DC.M6.2 Mitochondrial Respiration
DC.M5.9 Protein Synthesis
DC.M5.10 Mitochondrial Respiration
DC.M5.1 Inflammation
DC.M4.3 Protein Synthesis
DC.M3.5 Cell Cycle
DC.M3.2 Inflammation
0
.5
1
Effect size
−log10(q)
0.0
2.5
5.0
7.5
10.0
Direction
up
N
down
